# Production of Isolated Giant Unilamellar Vesicles under High Salt Concentrations

**DOI:** 10.3389/fphys.2017.00063

**Published:** 2017-02-13

**Authors:** Hannah Stein, Susann Spindler, Navid Bonakdar, Chun Wang, Vahid Sandoghdar

**Affiliations:** ^1^Friedrich-Alexander University Erlangen-NurembergErlangen, Germany; ^2^Max Planck Institute for the Science of LightErlangen, Germany

**Keywords:** giant unilamellar vesicle, lipid membrane, model membrane system, electroformation, swelling, double emulsion, microfluidic jetting, micropipette aspiration

## Abstract

The cell membrane forms a dynamic and complex barrier between the living cell and its environment. However, its *in vivo* studies are difficult because it consists of a high variety of lipids and proteins and is continuously reorganized by the cell. Therefore, membrane model systems with precisely controlled composition are used to investigate fundamental interactions of membrane components under well-defined conditions. Giant unilamellar vesicles (GUVs) offer a powerful model system for the cell membrane, but many previous studies have been performed in unphysiologically low ionic strength solutions which might lead to altered membrane properties, protein stability and lipid-protein interaction. In the present work, we give an overview of the existing methods for GUV production and present our efforts on forming single, free floating vesicles up to several tens of μm in diameter and at high yield in various buffer solutions with physiological ionic strength and pH.

## Introduction

With a high complexity in composition and shape, cellular membranes act as dynamic and flexible barriers separating the cell from the environment and enclosing internal compartments from the surrounding media. The large number of different lipids and membrane proteins result in unique membrane environments with highly specific functions in a cell (Coskun and Simons, 2011).

Single membrane components can diffuse freely within the membrane, and their diffusional behavior depends on the overall membrane composition (Dietrich et al., 2001; Eggeling et al., 2009), the surrounding medium (Böckmann et al., 2003; Wang et al., 2012), the underlying cytoskeleton (Kusumi et al., 1993; Mueller et al., 2011) and the membrane shape (Quemeneur et al., 2014). Studying the motion and interaction of lipids can link changes in the diffusional behavior to the overall membrane organization and therefore to specific membrane functions. However, investigating specific phenomena in the complex and dynamic membrane of a living cell poses a challenge since it is influenced by many, partly unknown processes. Therefore, it is helpful to reduce the complexity of the cellular membrane to that of basic lipid layers. Quantitative analyses and gradual increase of the complexity of the system would then allow one to draw conclusions about more complex behavior, e.g., in cellular membranes.

**Figure 1:**
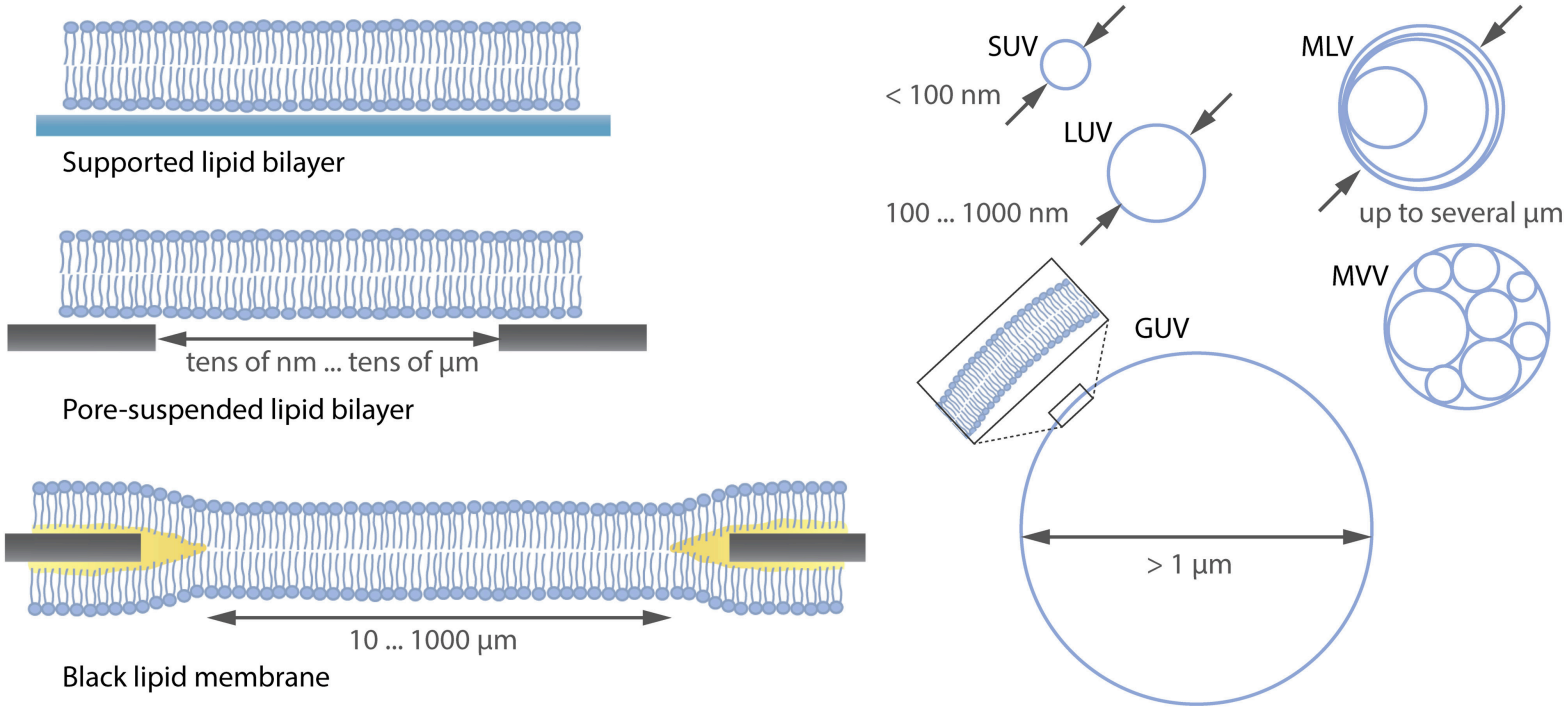
**Overview (not complete) over lipid membrane model systems ranging from flat lipid bilayers to differently sized liposomes**. SUV, small unilamellar vesicle; LUV, large unilamellar vesicle; MLV, multilamellar vesicle; MVV, multivesicular vesicle; GUV, giant unilamellar vesicle.

Many model systems for studying the lipid membrane are available under various conditions (Czogalla et al., 2014) as shown in Figure 1. For instance, supported lipid membranes offer an important tool because the lipid composition of the membrane can be adjusted precisely and proteins can be incorporated if a spacer such as a polymer cushion separates the membrane from the solid substrate (Wagner and Tamm, 2000). Moreover, they are extremely stable and enable the application of various biophysical investigation methods due to their flat geometry. Although the lower leaflet of the bilayer is expected to be 1-2 nm separated from the substrate by an aqueous layer (Czogalla et al., 2014), there is still an impact of the substrate on the diffusion of the membrane components (Przybylo et al., 2006; Hsieh et al., 2014). To overcome substrate limitations, “black lipid membranes” (Mueller et al., 1962) or pore-suspending membranes (Hennesthal and Steinem, 2000; Hennesthal et al., 2002; Heinemann and Schwille, 2011) can be used. However, also these systems can be affected by artifacts. Black lipid membranes show a reduced stability, and oil residues remain in the bilayer, influencing diffusion (Honigmann et al., 2010). Pore-suspending membranes experience edge effects caused by the small size of the pores (up to few μm) considering the diffusion coefficient of lipids to be in the order of few μm^2^/s. Giant unilamellar vesicles (GUVs) are the most promising alternatives for avoiding substrate effects.

GUVs are unilamellar vesicles consisting of a lipid bilayer (see Figure 1) with a diameter >1 μm. Their size is comparable to that of eukaryotic cells, thus mimicking the same lipid reservoir and membrane curvature (Fenz and Sengupta, 2012). Moreover, due to their large size they can be easily observed with optical microscopy and allow for investigations at single-vesicle level compared to using the so-called large unilamellar vesicles (LUVs) or small unilamellar vesicles (SUVs), where averaging over many vesicles is needed (Wieprecht et al., 2000; Ladokhin et al., 2010). The stability, precise adjustment of the lipid composition, the incorporation of proteins (Jørgensen et al., 2016) and the internal formation of an underlying cytoskeleton (Tsai et al., 2011) makes it possible to study cellular components in a well-controlled *in vitro* membrane.

The first protocols for GUV formation used water as growth medium (Reeves and Dowben, 1969; Angelova and Dimitrov, 1986), and GUVs were imaged using phase contrast microscopy. Today, in the majority of the GUV formation protocols a highly concentrated sucrose solution (typically between 100 and 300 mM) is used instead (Przybylo et al., 2006; Tareste et al., 2008; Fenz et al., 2009; Roux et al., 2010; Bi et al., 2013; Sezgin et al., 2015). By adding an equal-osmolar glucose solution after the formation, imaging with phase contrast is facilitated by the refractive index difference between sucrose and glucose. However, a concentrated sucrose solution can alter the properties of lipid membranes. Indeed, it has been shown that sucrose cross-links the head-groups of multiple lipids via hydrogen bonds, thus slowing down the lipid diffusion (Doeven et al., 2005; van den Bogaart et al., 2007).

In order to provide a more physiological environment for the lipid membrane, one can use a buffer solution which mimics the natural environment of a cell. The most important criteria of a buffer solution are stable pH value, chemical stability under variant conditions, and no membrane permeability of the buffer components (Good et al., 1966). A physiological amount of ions (around 300 mOsm/l) is essential for the stability of proteins (Beauchamp and Khajehpour, 2012) and for the interaction of biological molecules (Phillips et al., 2013). Since ions can also affect the diffusion of lipids (Böckmann et al., 2003; Wang et al., 2012), a physiological buffer should be used when studying diffusion in membranes. Although there are many methods to form GUVs under various conditions including ionic solutions (Akashi et al., 1996; Estes and Mayer, 2005a; Montes et al., 2007; Pott et al., 2008; Horger et al., 2009; Walde et al., 2010; van Swaay and deMello, 2013; Weinberger et al., 2013), the formation of vesicles larger than 20 μm and at high yields still poses a challenge if one insists on compatibility with buffer solutions of physiological ionic strength and lack of residues such as oil or polymers. Moreover, detachment for GUVs grown on a substrate becomes increasingly difficult with increasing ion concentration, but is often not adequately addressed. Here, we present different methods to produce free floating, single GUVs with diameter up to 100 μm in buffer solutions with physiological ionic strength.

## Natural swelling

**Figure 2:**
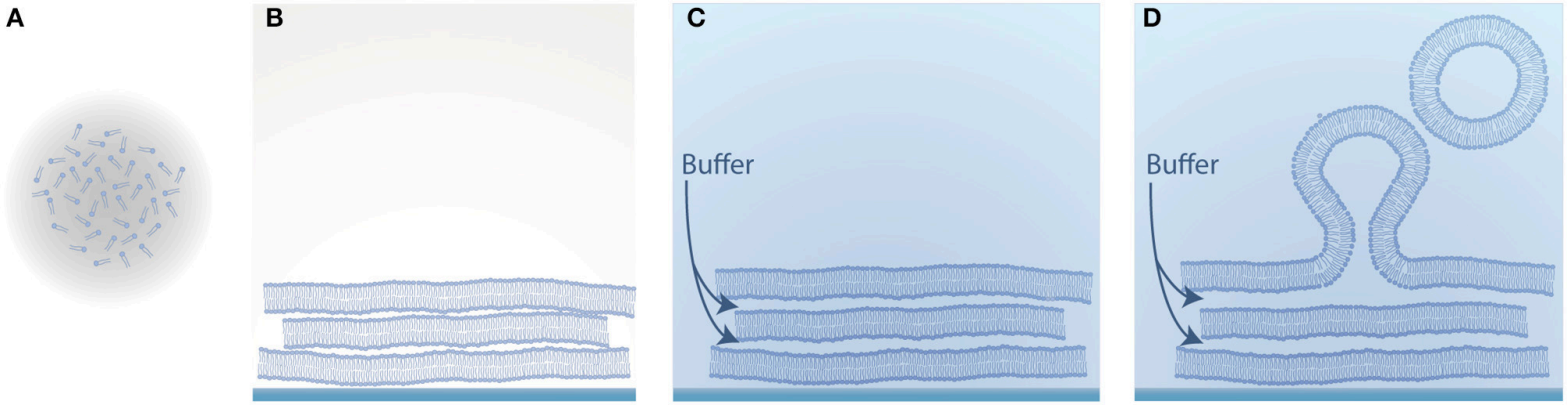
**Schematic illustration of vesicle formation by natural swelling. (A)** Lipids dissolved in an organic solvent; **(B)** Evaporation of the solvent and self-assembly of the amphiphilic lipid molecules into several stacks of bilayers; **(C)** Hydration of the dried lipid film with aqueous solution; **(D)** Swelling of the lipid film into vesicles.

One of the earliest approaches to form GUVs is natural swelling and was introduced by Reeves and Dowben (1969). Vesicles grow from prehydrated layers of stacked lipid bilayers due to a combination of osmotic pressure, electrostatic interactions and the hydrophobic effect (Tsumoto et al., 2009), as illustrated in Figure 2. Here, lipids dissolved in chloroform are deposited on a solid substrate and as the solvent evaporates, the amphiphilic structure of lipids leads to the clustering of several stacks of bilayers. By adding a buffer solution, vesicles can be obtained after several days.

An important driving force for the swelling process is the flow of the aqueous solution in between the bilayer stacks (Tsumoto et al., 2009) caused by osmotic pressure. Due to the irregularity of the bilayer stacks on the glass substrate caused by the lipid deposition, the hydrophobic core of the lipid bilayer can be exposed to the aqueous environment. This energetically unfavored state forces the bilayer to enclose the vesicles in order to shield the hydrophobic core (Politano et al., 2010). In order to improve the osmotically driven flow, sucrose (Tsumoto et al., 2009) can be deposited between the lipid layers. Furthermore, an increased distance between the lipid layers can lead to a more effective influx of the buffer solution in between the bilayer stacks (Akashi et al., 1996). The spacing is caused by both thermal fluctuations and electrostatic repulsion of the lipid head-groups. Therefore, adding a certain amount of charged lipids can help to increase the GUV yield (Akashi et al., 1996).

GUV formation by natural swelling has the big advantage that it is mild and does not interfere with the oxidization of lipids. Nevertheless, the method is limited in several ways. First, natural swelling requires long time scales on the order of days (Rodriguez et al., 2005). Second, a large amount of multilamellar vesicles and lipid debris are formed and only a small fraction of the obtained vesicles are GUVs. These GUVs often show a bad membrane quality with internal tubules and attached small vesicles (Rodriguez et al., 2005). Third, a reasonable number of GUVs in physiological buffer can only be achieved with a fraction of charged lipids (Akashi et al., 1996). Furthermore, it has been shown that the lipid composition of vesicles in the same sample can vary up to 10-fold due to the inhomogeneity of the lipid film on the substrate (Larsen et al., 2011). It should be noted that this applies for every method where lipids are spread on a solid substrate, as is also the case for electroformation and gel-assisted swelling.

## Electroformation

In order to enhance the swelling process, Angelova and Dimitrov introduced GUV formation by a DC electric field in 1986 (Angelova and Dimitrov, 1986). They improved the method by applying an AC field which leads to a higher GUV yield due to the periodic redistribution of charges (Dimitrov and Angelova, 1987). With this method, large amounts of cell-sized vesicles can be obtained within few hours in distilled water or sucrose. Here, the lipids are deposited on two electrodes, and the swelling of the dried lipid film into vesicles is enhanced by an electric field due to an interplay between electrostatic interaction, redistribution of bilayer counter ions, membrane surface and line tension changes, as well as electro-osmotic effects (Dimitrov and Angelova, 1987). Upon application of an electric field, the lipids follow the alternating electric field depending on their net charge. The non-covalently bound counter ions of the lipids redistribute between the bilayers following the electric field. Furthermore, the electric field induces weak dipoles in the solution, causing a flow and membrane fluctuations, which lead to the separation of the bilayers if the electro-osmotic effects overcome the van der Waals attraction between the bilayers (Politano et al., 2010). In this case, the water influx in between the bilayer stacks is increased and the separated membrane bulges out. The resulting membrane buds are densely packed on the surface of the lipid layers. Due to the dense packing and the influence of the electric field, the membrane buds fuse which can be observed during the formation process. Recently, it has been shown that enclosed and detached vesicles do not fuse during electroformation (Micheletto et al., 2016). By decreasing the frequency of the AC field, the swollen vesicles follow the slow change of the field and detach from the surface, forming free floating GUVs (Politano et al., 2010).

### Electroformation on Pt wires

**Figure 3:**
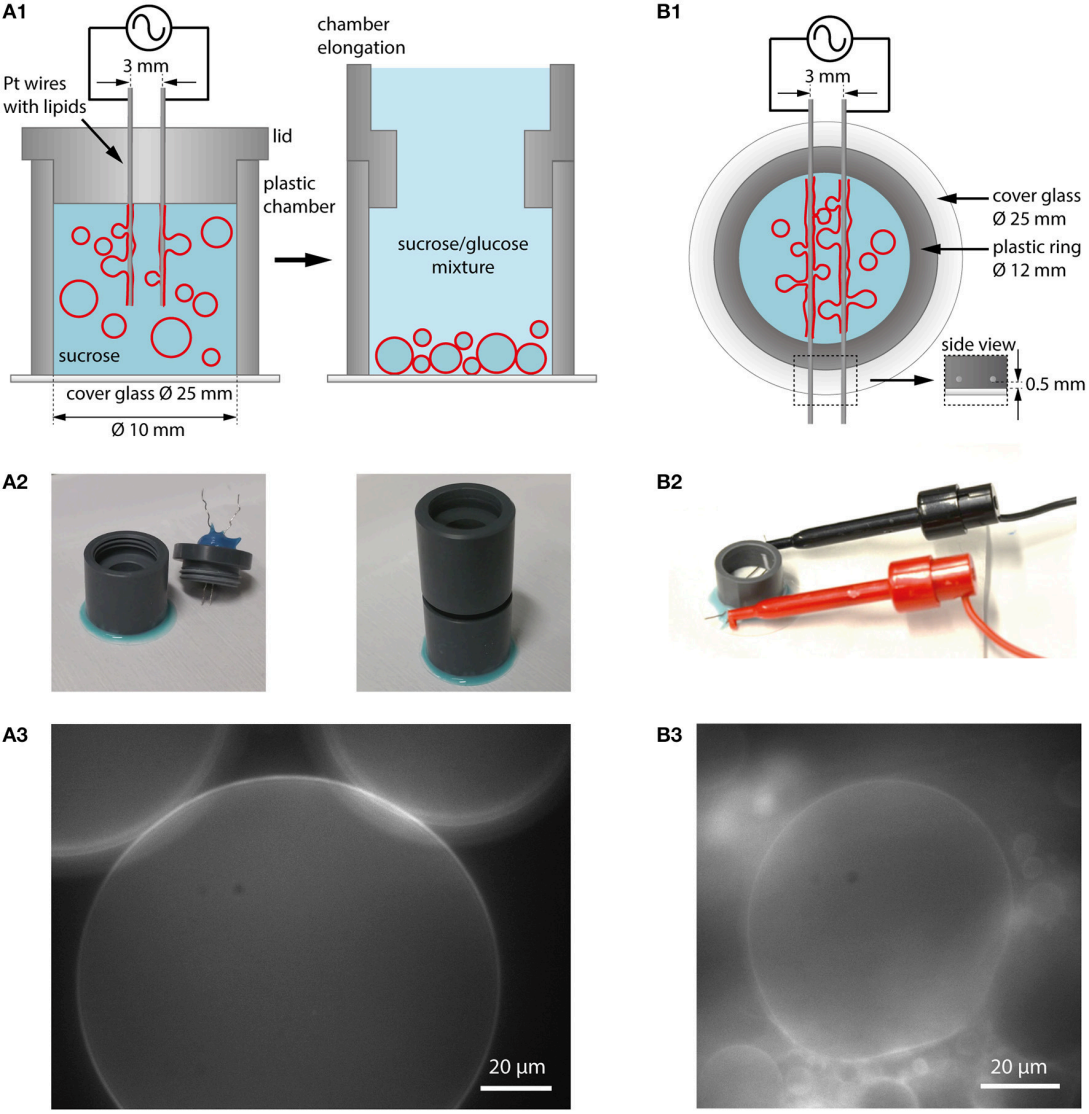
**Electroformation using Pt wires as electrodes. (A1)** Schematic illustration of a vertical chamber geometry (cross section). The chamber consists of a plastic ring glued to a cover glass. The chamber is filled with aqueous solution (e.g., sucrose) and a lid with two Pt wires coated with lipids is placed on top. Application of an AC electric field leads to GUV swelling and detachment. After formation, the lid can be replaced with a chamber elongation. In case of sucrose as swelling solution, glucose can be added to the observation buffer which leads to sinking of the GUVs to the bottom for observation and easy harvesting. **(A2)** Photographic images of the vertical chamber. **(A3)** GUVs in sucrose/glucose solution at the bottom of the growing chamber (lipid composition: DOPC with 0.02 mol-% Atto532-DOPE). **(B1)** Schematic illustration of a horizontal chamber geometry (top view). A plastic ring is glued onto a cover glass. The platinum wires with the lipids are inserted into a plastic ring very close to the edge of the ring to be in small distance to the glass (see inset labeled with side view). **(B2)** Photographic image of the horizontal chamber. **(B3)** Observation of GUV growth during electroformation in sucrose (lipid composition: DOPC with 0.02 mol-% Atto532-DOPE).

Platinum (Pt) wires were used as electrodes in first electroformation experiments (Angelova and Dimitrov, 1986), which can be positioned either vertically or horizontally in a formation chamber made of teflon or polyvinyl chloride (PVC). In Figure 3, our implementation of both geometries is shown.

Lipids dissolved in chloroform or another solvent mixture are spread onto the wires with a microsyringe, and the solvent is evaporated using a desiccator. After assembling the chamber and adding the swelling solution, both wires are connected to a function generator. In the vertical configuration, the wires are mounted into the lid of the chamber and can be easily removed after the formation procedure. When using sucrose as swelling solution, the GUVs can be sunk to the bottom of the chamber by extending the chamber from the top and adding a solution with a lower density such as glucose (see Figures 3A1,A2). Choosing a slightly lower osmolarity for glucose compared to sucrose (e.g., 275 mM glucose and 300 mM sucrose) leads to water influx and the reduction of large membrane fluctuations. By attaching a cover glass to the bottom of the chamber using a removable glue (e.g., using Picodent Twinsil), the GUVs can be directly observed (Figure 3A3) and harvested for further experiments with a pipette tip (cut at the end to have a larger opening and to limit shear stress on the GUVs). The horizontal configuration allows for direct visualization of the process through the cover glass when positioning the wires within the working distance of the microscope objective.

### Electroformation on ITO coated glasses

**Figure 4:**
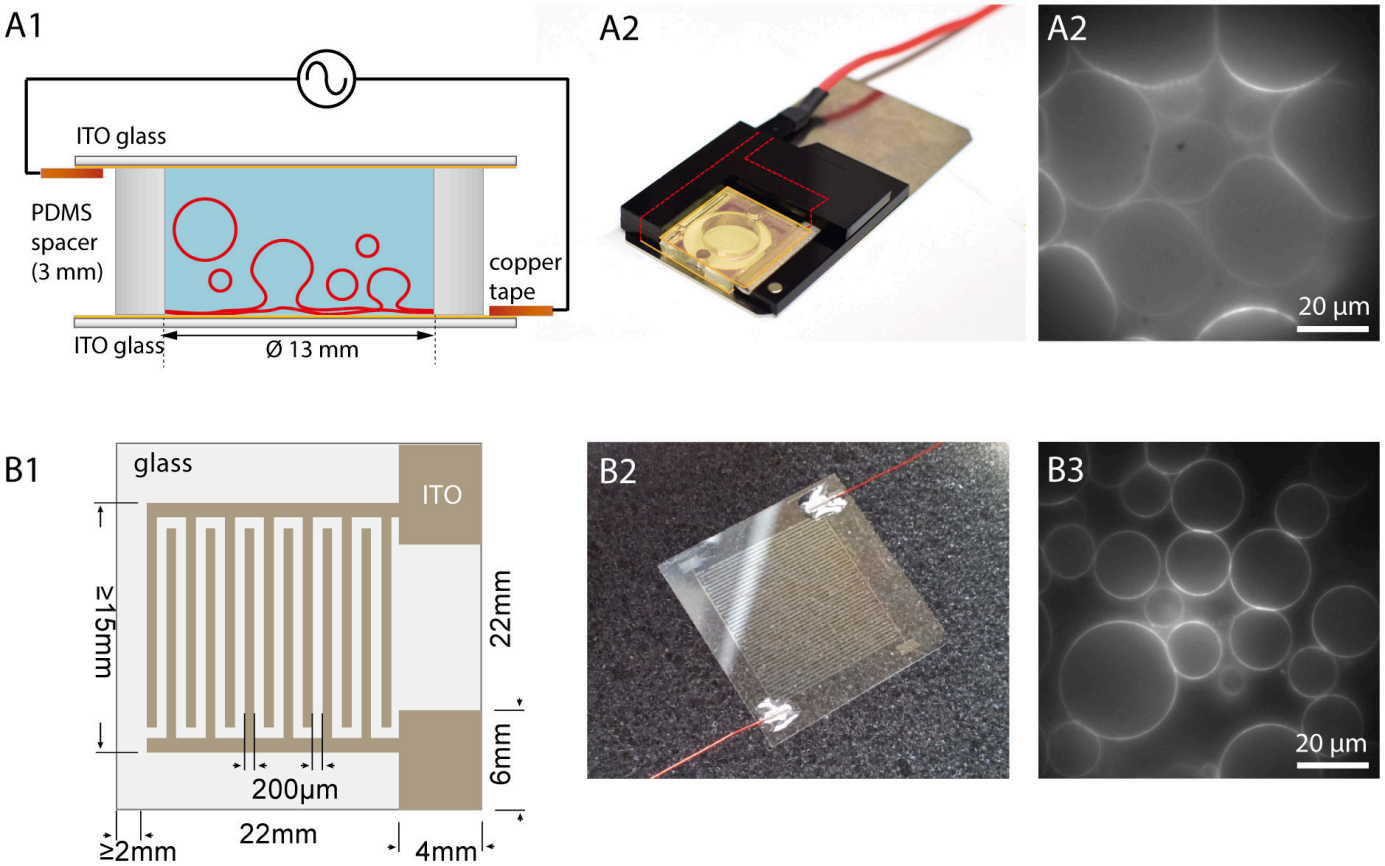
**Electroformation using ITO coated glasses. (A1)** Schematic illustration of an ITO chamber. Two ITO coated glasses with conducting sides facing each other are separated by a spacer. Connecting the ITO layers via copper tape to a function generator leads to swelling of the GUVs. **(A2)** Photographic image of an ITO chamber. A plastic frame with magnets holds the chamber closely together. **(A3)** Observation of GUV growth during electroformation in sucrose (lipid composition: DOPC with 0.02 mol-% Atto532-DOPE). **(B1)** Schematic illustration of a cover glass with interdigitated ITO electrodes with 200 μm spacing. **(B2)** Photographic image of the glass with interdigitated ITO electrodes connected with wires to a function generator. **(B3)** Observation of GUVs grown on the interdigitated electrodes (lipid composition: DOPC with 0.02 mol-% Atto532-DOPE).

Another possibility to observe the GUVs and the formation process over the whole area is to use flat electrodes, e.g., consisting of glasses coated with indium tin oxide (ITO) (Angelova et al., 1992) or gold (Niri et al., 2009). Here, besides direct optical access, a larger GUV yield can be obtained due to the larger surface area. The principle is shown in Figure 4. Two ITO-coated cover glasses separated with a sticky spacer, e.g., made from PDMS, are connected via copper tape to a function generator. Before assembly, the lipid solution is spread on the conductive site of one or both of the ITO glasses. After evaporating the solvent, the spacer is placed on one glass and filled with the swelling solution. By carefully placing the second ITO glass on top, the chamber is closed. To avoid leakage of the chamber, two clamps can be used to press the two ITO glasses together. In our configuration, we use two plastic frames with conductive copper tape facing the ITO and use magnets to fix them to a magnetic plate. The contacts are immediately connected to a function generator. After the formation process, the upper glass is carefully removed, and the GUVs can be harvested.

In 2013, Bi et al. introduced the usage of coplanar electrodes (Bi et al., 2013). Here, photolithography and electrochemistry are used to produce interdigitated electrodes on an ITO coated glass, as shown in Figures 4B1,B2. Placing a ring on the top creates a chamber, which can be filled with the swelling solution. A second glass can be used to close the chamber although this is not necessary for the formation procedure. An open geometry allows for access and manipulation during the formation process. Moreover, it was found that smaller electrode widths lead to bigger GUVs due to higher field strengths (Bi et al., 2013). Hence, this method offers the possibility to tune the GUV size by adjusting the electrode size within a narrower range compared to planar ITO coating (see Figure 4B3).

### Frequency protocols for different swelling solution

**Figure 5:**
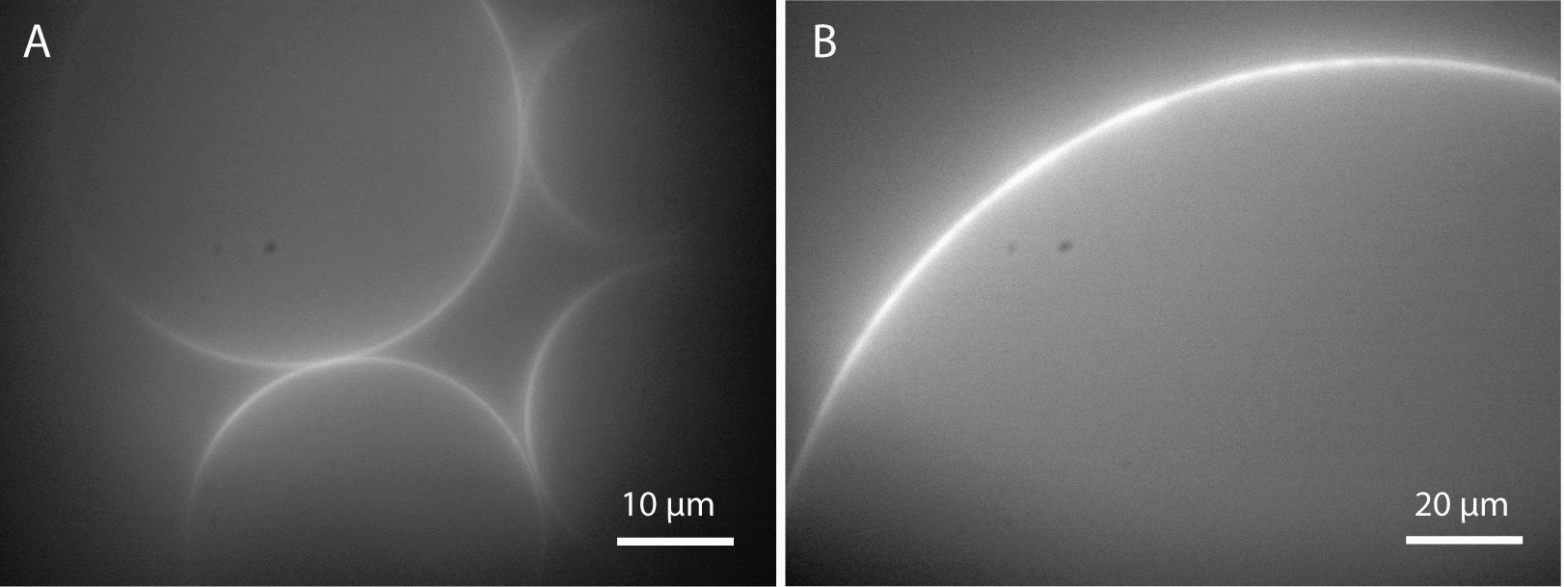
**GUVs prepared by electroformation: (A)** in deionized water and **(B)** in 300 mM sucrose after applying 10 Hz and 2 Vpp for 2 h (lipid composition: DOPC with 0.02 mol-% Atto532-DOPE).

GUVs in the range of 50–100 μm or even larger can be formed in water or in sucrose solution by applying an AC voltage around 2–3 V_pp_ for 2 h at 10 Hz (Herold et al., 2012). Optimization of the frequency protocol can lead to an increased GUV yield as shown by Li et al. for GUV formation in pure water (Li et al., 2016). Figure 5 shows GUVs prepared in deionized water and an example of a supergiant vesicle of 220 μm diameter prepared in 300 mM sucrose. Detachment of the GUVs from the electrodes can be achieved by lowering the frequency to 2 Hz for around 30 min. At this low frequency, the vesicles attached to the electrodes can follow the electric field (see Movie M1) and tear off from the substrate. However, buffer solutions with physiological ionic strength lower the yield of giant vesicles dramatically (Politano et al., 2010), mainly because of the electric field shielding by the ions of the buffer solution (Politano et al., 2010). One approach to overcome this limitation was published by Estes and Mayer (2005a). They form big vesicles in a glycerol-containing buffer by electroformation and exchange the solution inside and outside of the vesicles with an isotonic physiological buffer using a home-built flow chamber. Although a high amount of cell-sized vesicles is produced with this technique, it cannot be excluded that the remnants of the swelling solution alter the diffusion behavior of the membrane. In order to form vesicles in physiological buffer by electroformation, many voltage-frequency protocols have been published (e.g., Montes et al., 2007; Pott et al., 2008). By increasing the frequency of the AC field, the shielding of the electric field can be leveled out because the ions do not compensate the alternating electric field that fast. Hence, by adjusting the voltage-frequency protocols, GUV formation via electroformation can be adapted to buffers with different ionic strengths and osmolarities.

In this work, we optimized the GUV yield for three different buffers using a lipid mixture of 1 mg/ml DOPC and 0.02 mol-% Atto532-DOPE: the low ionic Tris buffer (10 mM Tris-HCl and 1 mM EDTA), a buffer containing 10 mM HEPES and 150 mM NaCl to which we refer to as “Bilayer buffer,” and PBS (150 mM Sodium phosphate and 150 mM NaCl). We tested various electroformation protocols in the range of 1–6 V_pp_ and 10–500 Hz with ITO chambers as well as platinum wires.

**Figure 6:**
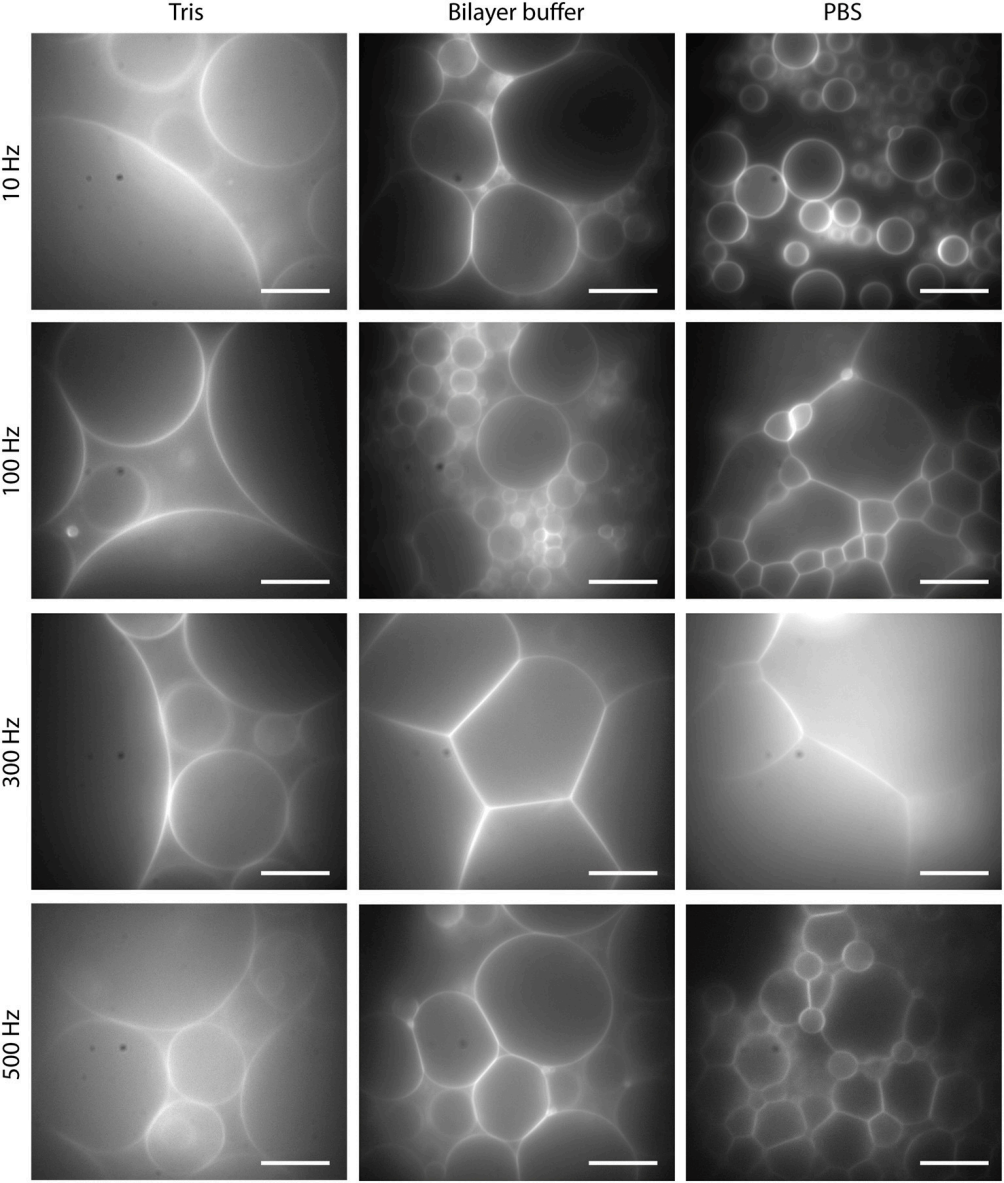
**GUVs formed by electroformation for 2 h at 2 V_**pp**_ and different frequencies for Tris buffer, Bilayer buffer and PBS**. Lipid composition: DOPC and 0.02 mol-% Atto532-DOPE. Scale bars: 10 μm.

For both ITO and platinum, a large number of GUVs were obtained for all three buffers in a frequency range between 10 and 500 Hz and amplitudes between 1 and 3 V_pp_. Increasing the voltage up to 6 V_pp_ did not increase the GUV size and yield further. Usually, the size distribution is quite broad. At 10 Hz, GUVs bigger than 30 μm can only be obtained in Tris buffer due to the low ion concentration. We identified 300 Hz and 2 V_pp_ as the best suited in terms of GUV yield with sizes larger than 30 μm for all three buffers, see Figure 6. In Tris buffer with the lowest ion content, GUVs up to 150 μm or even larger can be easily obtained. Lowering the frequency to 2 Hz for around 30 min leads to a high yield of free floating vesicles in Tris. However, the yield of GUVs larger than 30 μm in buffer solutions with physiological ionic strength such as Bilayer buffer or PBS is lower and especially the detachment is challenging due to the hemispherical shape of most of the GUVs. Detachment by lowering the frequency to 2 Hz leads only to a low number of free floating GUVs due to the high ion concentration and the shielding of the field. We observed that GUVs start to follow the field at 2 Hz only at voltages above 5 V_pp_ although even then the amount of detached vesicles is fairly low. Gentle pipetting did not increase the amount of detached GUVs significantly.

### Influence of lipid spreading

It is pointed out in the literature that the thickness of the lipid layer is important for a high GUV yield. The appropriate thickness is estimated to be in the range from 5 to 10 lipid bilayers (Angelova and Dimitrov, 1986; Angelova et al., 1992). To achieve homogeneous spreading of the lipid solution on the substrate at the optimal thickness, Estes and Mayer use spin coating of the lipids on ITO substrates (Estes and Mayer, 2005b). However, the amount of lipids needed to cover the whole surface is quite large. Moreover, we did not observe a clear improvement in the GUV yield when spin coating the lipids compared to spreading with a microsyringe. In fact, a non-uniform deposition and therefore more layer defects may lead to a better water influx, facilitating the GUV growth. We, thus, tested also other methods, such as drop formation or strip-like spreading for depositing lipids on ITO. Nevertheless, we could not observe an improvement compared to spreading the lipid solution with a microsyringe over the whole area.

### Drawbacks

**Figure 7:**
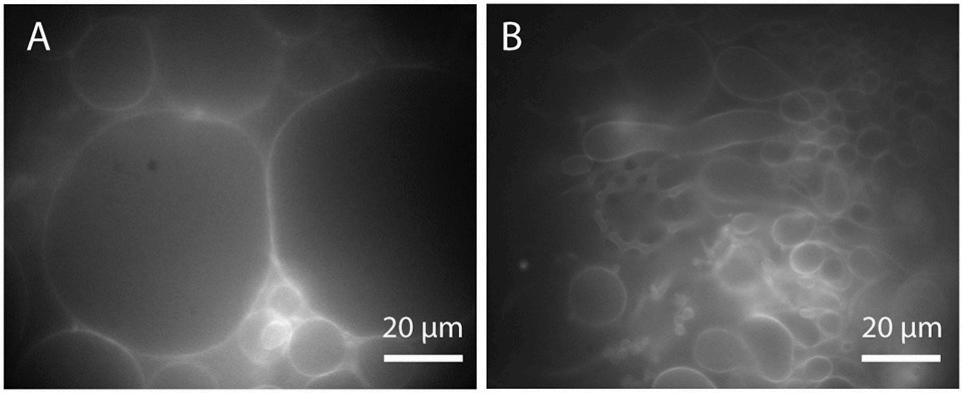
**Effect of ITO layer degradation on the GUV yield (Tris buffer, 300 Hz, 2Vpp): (A)** GUVs formed on new and **(B)** on used and discolored ITO glasses (lipid composition: DOPC with 0.02 mol-% Atto532-DOPE).

Easy implementation and high efficiency make electroformation a widespread method for GUV production. However, this technique also has some drawbacks. Although electroformation protocols can be adapted to various conditions, the GUV yield in physiological buffer solutions remains fairly low. Furthermore, only a small amount of charged lipids can be used for GUV formation by electroformation (Rodriguez et al., 2005). The optimization of electroformation protocols to higher ratios of charged lipids (Montes et al., 2007) only leads to small GUVs with a low yield. Another drawback of electroformation is that the population of GUVs formed from a lipid mixture is very heterogeneous in terms of their lipid composition. Besides the heterogeneity caused by the lipid deposition (Larsen et al., 2011), some lipids react differently to the applied electric field depending on their charge and are incorporated in the GUVs to a different extent. Therefore, the lipid composition of GUVs in a single formation chamber can vary (Veatch et al., 2004). As a consequence, the interpretation and comparison of experiments, in which the lipid composition is crucial, is a major challenge. Furthermore, we observed that the conductive substrate discolors after many times of electroformation. This indicates that electrode material dissolves in solution when a voltage higher than the standard electrode potential is applied. Since ITO has a fairly low electrochemical constant (indium −0.14 V and tin +0.34 V) (Lide, 2005), materials like gold (+ 1.4 V) or platinum (+1.2 V) (Lide, 2005) are more suitable for electroformation although it is hard to avoid the effect completely since most protocols require voltages higher than the electrochemical constants to maximize the GUV yield. Indeed, using ITO-coated glasses, we obtained a decrease in the GUV yield after frequent usage of the substrates, see Figure 7. Annealing for 20 min at 150°C, as recommended in the literature (Herold et al., 2012), can help to restore the quality of the ITO coating after few times of usage, but not indefinitely in our case. A possible reason for this might be that the degradation of the ITO coating is enhanced in saline solution compared to deionized water as used in Herold et al. (2012). In contrast, platinum wires can be cleaned effectively by electrolysis of 1 M NaCl solution (Aimon et al., 2011, supporting text S2) so that the GUV yield is not affected.

Another important disadvantage of electroformation is the impact of the electric field on the oxidation of lipids (Zhou et al., 2007). Especially polyunsaturated lipids, which play a central role in many biological membranes, are prone to peroxidation by electric fields in a dose dependent manner. It has been shown that the standard electroformation protocol with 2 V for 2 h is sufficient to cause oxidation of lipids (Zhou et al., 2007). The resulting chemical changes in the lipid structure can lead to dramatically altered behavior of the lipids in the GUV membrane such as reduced membrane stability (Zhou et al., 2007), increased membrane permeability (Sankhagowit et al., 2014; Runas and Malmstadt, 2015), or even altered raft behavior (Ayuyan and Cohen, 2006).

## Gel-assisted swelling

### Agarose-assisted swelling

**Figure 8:**
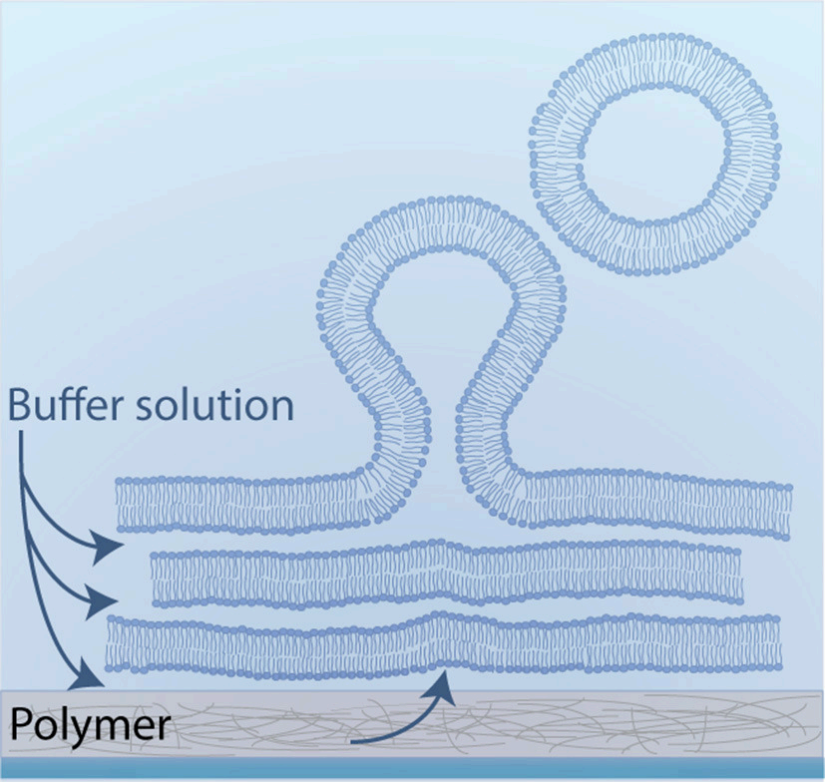
**Schematic illustration of polymer-assisted swelling:** Upon hydration of a dried lipid film, vesicles swell at an enhanced rate due to an accelerated buffer flow from below.

Horger et al. developed GUV formation by polymer-assisted swelling on an agarose gel (Horger et al., 2009). The aim of polymer-assisted swelling is to increase the buffer influx without any disruption of the membrane. Therefore, the lipids are deposited on a porous polymer layer to enhance the buffer flow from below (Figure 8). This increased flow leads to the rapid formation of unilamellar vesicles over a wide range of lipid compositions in various buffer solutions (Horger et al., 2009). The rate of vesicle growth is high enough that the crowded vesicles fuse to form big GUVs. Most of the obtained vesicles are unilamellar without any visible defects such as attached lipid debris or internal tubules.

The drawback of using agarose as polymer is that the deposited lipids penetrate the porous agarose film, and that the swollen vesicles contain a high amount of agarose inside (Horger et al., 2009; Lira et al., 2014). The enclosed agarose is autofluorescent, which can bias fluorescent-based studies. Furthermore, agarose dissolves in solution and interacts with the lipids in the GUV membrane. This can lead to altered mechanical properties of the vesicles and increases the permeability of the membrane (Lira et al., 2014). We note that even though the mobility of lipids in the GUV membrane is tested with FRAP (fluorescence recovery after photobleaching) (Horger et al., 2009), systematic effects on the diffusion behavior of the lipids cannot be excluded.

### PVA-assisted swelling

To improve the method of swelling on a porous substrate, Weinberger et al. introduced polyvinyl alcohol (PVA) as the underlying polymer film (Weinberger et al., 2013). Aside from its simplicity (for details see Materials and Methods section), the main advantage of this method is that the swollen vesicles do not contain any remnants of the polymer inside since the lipids do not penetrate the PVA film, but rather assemble on top of the matrix to several stacks of lipid bilayers. Furthermore, PVA does not dissolve in solution at room temperature and thus there is no detectable PVA impurity in the membrane. No altered mechanical properties or altered diffusional behavior has been reported for GUVs grown on a PVA film (Weinberger et al., 2013), but has not been tested systematically so far.

**Figure 9:**
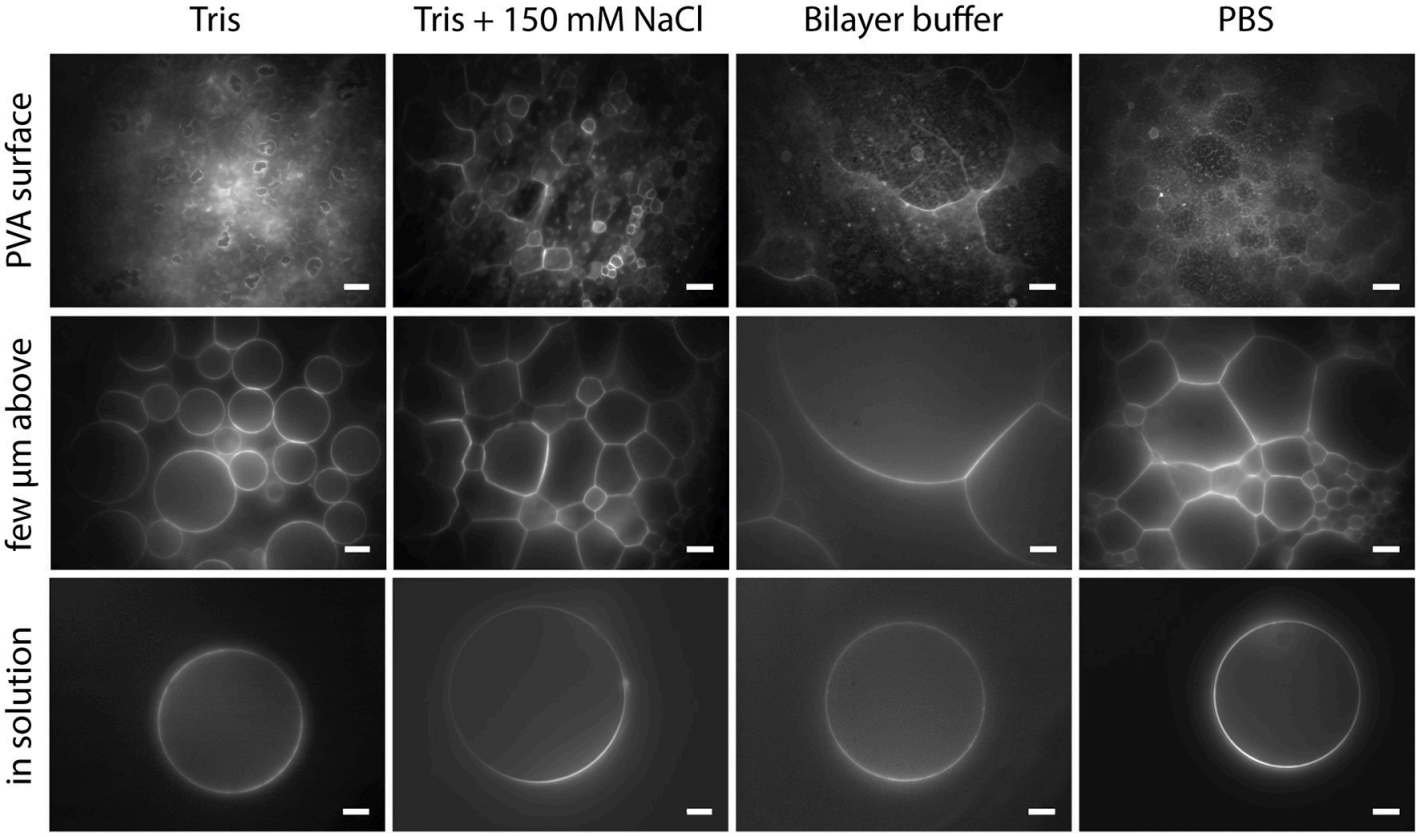
**GUVs formed by PVA-assisted swelling in Tris buffer, Tris buffer + 150 mM NaCl, Bilayer buffer and PBS (left to right):** Vesicles attached to the lipid film focused on the lipid film **(upper row)**, a few micrometers above **(middle a row)** and detached vesicles in solution **(lower row)**. Lipid composition: DOPC and 0.02 mol-% Atto532-DOPE. Scale bars: 10 μm.

With PVA-assisted swelling it is possible to obtain GUVs up to 100 μm or even bigger in various buffer solutions within minutes, as shown in Figure 9 (see also Movie M2). In Tris buffer, closed vesicles grow spatially separated from each other and can be easily detached from the lipid film by tipping on the bottom of the growing chamber (Figure 9, column 1). By adding a physiological amount of salt to the Tris buffer, the vesicles swell densely packed and stay attached to the lipid film as half spheres (Figure 9, column 2). We obtained this dense packing and semicircular vesicle shape also in bilayer buffer and PBS (Figure 9, column 3 and 4 and Movie M2).

These different properties of the swelling process may be caused by the interaction of the buffer solution with the lipid membrane. For instance, it has been shown that sodium chloride, Tris or Hepes reduce the bending rigidity of a lipid bilayer to a different extent (Bouvrais et al., 2014). Additionally, the spacing between the bilayer stacks is increased with a higher salt or buffer concentration (Koerner et al., 2011). These influences of the buffer molecules may offer an explanation for the different swelling properties of GUVs in various buffer environments.

**Figure 10:**
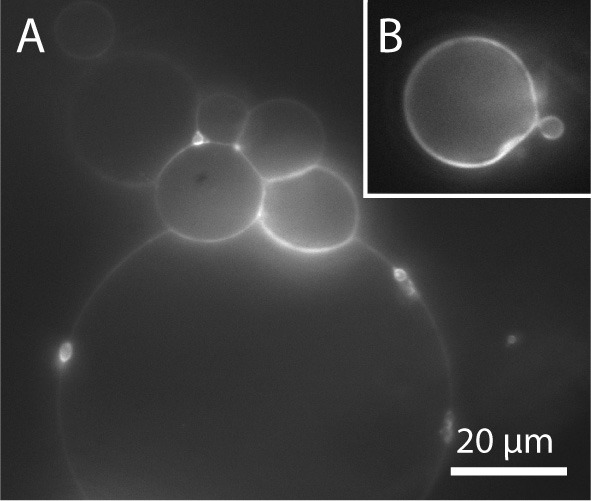
**GUVs prepared by PVA-assisted swelling in Bilayer buffer and transferred into an observation chamber. (A)** Vesicle cluster detached by tipping against the growing chamber. **(B)** Vesicle with membrane defects detached by sonication. Lipid composition: DOPC and 0.02 mol-% Atto532-DOPE.

In order to facilitate the detachment of densely packed and incomplete vesicles, a small amount of lipids should be deposited on the PVA film (around 5 μl for an area of 22 mm diameter). In this fashion, a lower amount of vesicles grows, and they can be spatially separated so that single vesicles can be detached by gently pipetting up and down 200 μl buffer solution close above the glass surface. To transfer the GUVs into an observation chamber, we either pipette out the vesicles with a cut tip or rinse it into the observation chamber. With this cautious detachment, we can obtain a sufficient number of free floating vesicles in the range of 10–100 μm or more without visible membrane defects. In Tris buffer, also gentle tipping against the bottom or the side walls of the chamber helps to detach the GUVs. However, in the case of bilayer buffer or PBS this leads to the detachment of densely packed GUV clusters as can be seen in Figure 10A. Thus, this should be avoided. Weinberger and co-workers recommend gentle sonication to detach GUVs from the lipid film (Weinberger et al., 2013). We tested sonication for few seconds in continuous and pulsed mode with an ultrasonic bath (EMAG Emmi 40HC at 50% power). However, we observed an increased amount of defects such as increased membrane fluctuations as well as internal membrane tubules or small attached lipid debris (Figure 10B). Furthermore, mechanically stressed vesicles are rather small, presumably due to rupture during the harsh detachment process.

We tested different ways to deposit the lipids on the PVA film such as drops, stripes or snake-like patterns. We found that spreading the lipids uniformly with a microsyringe on the PVA film leads to a high swelling rate and to a maximal number of detached vesicles.

With PVA-assisted swelling, the formation of GUVs under various salt conditions results in a sufficient amount of free floating GUVs up to 100 μm or even more. The method is easy to implement and does not require any special equipment. In contrast to electroformation, PVA-assisted swelling is not limited to low salt concentration or lipids with a low number of charges. Therefore, lipid swelling on a PVA film is well suited to the formation of GUVs in various buffer conditions over a wide range of lipid compositions.

To improve the method even further and to make sure that absolutely no polymer dissolves during the swelling process, a cross-linked hydrogel can be used (López Mora et al., 2014). This also allows for regulating the GUV size to a certain extent because the pore size can be adjusted precisely by varying the density of the cross-linked polymer (López Mora et al., 2014).

## Microfluidics

Microfluidic approaches offer the ability to produce monodisperse GUVs at high throughput with various contents (van Swaay and deMello, 2013).

Funakoshi et al. introduced jetting as a microfluidic method for GUV formation in 2007 (Funakoshi et al., 2007). Here, the buffer solution is shot onto a pre-assembled lipid bilayer at a water-oil interface using a micronozzle or micropipette and GUVs are formed out of this bilayer. This method was improved by Stachowiak et al. by using a piezoelectric actuator of an inkjet printer (Stachowiak et al., 2009; Richmond et al., 2011). The size, the inner and outer solution as well as the lipid composition of the inner and outer leaflets can be adapted. However, a residual amount of oil needed to form the bilayer at the interface with a reservoir of dissolved lipids stays in the hydrophobic core of the membrane (Funakoshi et al., 2007; Kirchner et al., 2012). This can lead to altered mechanical properties and diffusional behavior in and across the membrane.

Weitz and co-workers developed another microfluidic approach in 2008 to produce monodisperse GUVs at a water-oil-water interface, the so-called double emulsion method (Shum et al., 2008). Here, water-in-oil-in-water droplets are formed, and solvents that are contained in the oil phase evaporate, thus forming GUVs out of the double emulsion droplets. However, also here some residual solvents can remain trapped within the bilayer (van Swaay and deMello, 2013), rendering the GUVs unsuitable for diffusion studies or domain formation (Arriaga et al., 2014).

**Figure 11:**
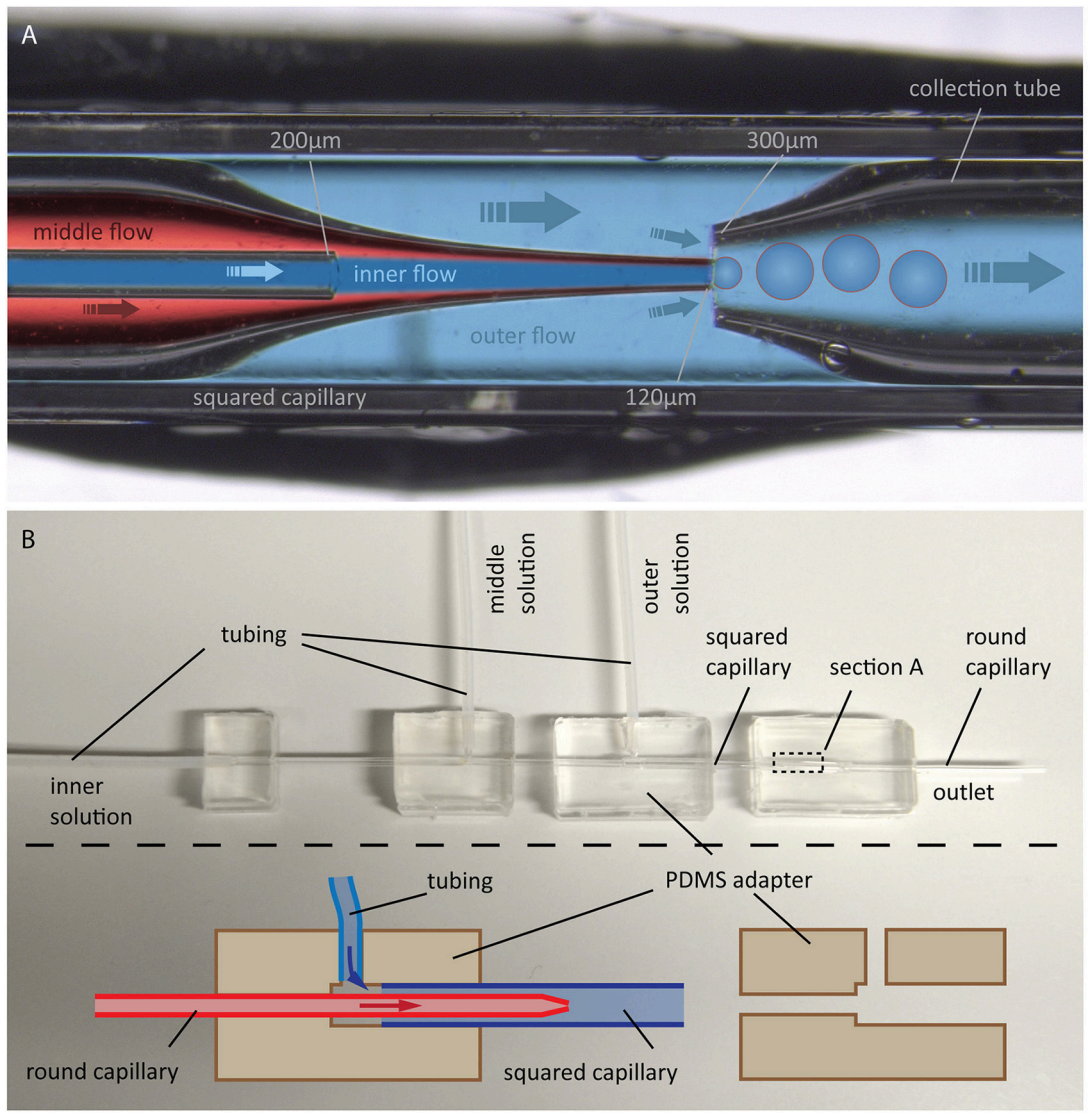
**GUV production by the double emulsion method. (A)** Photographic image with overlaid schematic illustration of GUV formation using double emulsion: Flow of an aqueous solution (inner flow) into an oil phase flow containing lipids (middle flow) leads to bulging at the tip of a tapered capillary. Introducing a flow of another aqueous solution (outer flow) in between the outer and middle capillary walls into another tapered capillary causes a tear-off resulting in double emulsion droplets, which are collected by the right capillary. **(B)** Photographic image of the whole configuration including a schematic illustration of the PDMS adapters for connecting the capillaries and for flow inlet of the different solutions.

In 2014, Arriaga et al. improved this method to form ultrathin shell double emulsion vesicles with minimized amount of residual solvents, thus allowing for microdomain formation (Arriaga et al., 2014). We followed this approach, as illustrated in Figure 11.

**Figure 12:**
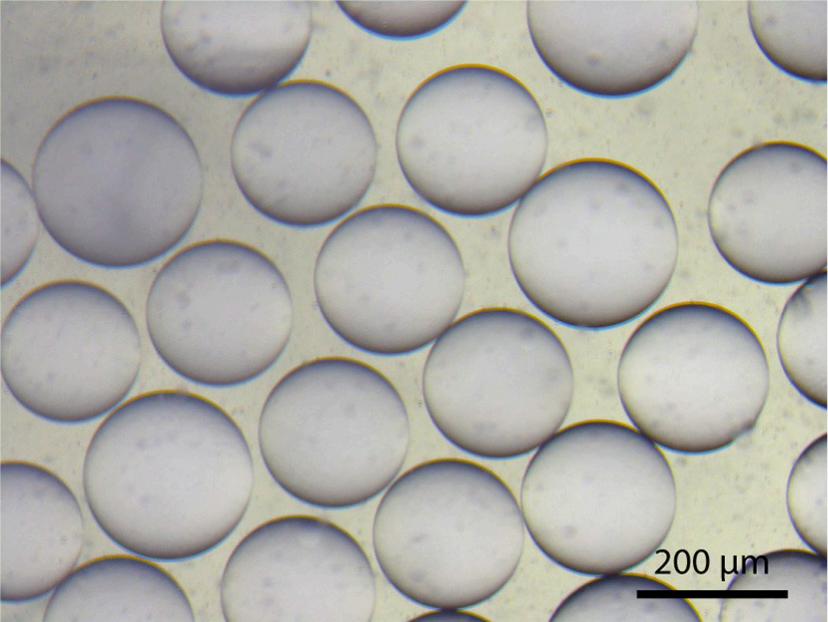
**DOPC-GUVs formed from double emulsion droplets**.

The device consists of two tapered round capillaries (outer diameter 1 mm), inserted into the opposite ends of a squared capillary (inner diameter 1.05 mm) to allow for precise alignment of their axes. Another smaller round capillary (outer diameter around 200 μm) is inserted into the left tapered capillary. The latter is treated (e.g., with n-octadecyl-trimethoxy silane) to make the surface hydrophobic and, thus, prevent wetting of the inner aqueous phase. Both the inner aqueous phase and the middle oil phase containing lipids dissolved in a chloroform/hexane mixture are injected. This leads to water-in-oil emulsion drops at the end of the left tapered capillary. By injecting the outer aqueous phase between the walls of the squared and left tapered capillary, double emulsion drops are formed. The drops are collected by the right tapered capillary, which is treated with, e.g., 2-(methoxy (polyethyleneoxy) propyl) trimethoxy silane. This renders the surface hydrophilic and prevents wetting of the middle oil phase on the capillary wall. The remaining oil in the hydrophobic core evaporates, and monodisperse unilamellar vesicles are formed within seconds. The size of the GUVs can be adjusted by the flow, which is controlled using a pressure controller or a syringe pump. Using this microfluidic capillary device we were able to produce GUVs of over 200 μm in diameter as shown in Figure 12. The inner solution contains poly(ethylene glycol) (PEG) and poly(vinyl alcohol) (PVA), while the outer solution is PVA (Arriaga et al., 2014). Such viscous solutions are needed for the stability of the GUVs. By using glycerol in the outer aqueous phase and a diblock co-polymer (Pluronic F68), the stability of the vesicles can even reach the order of several months (Teh et al., 2011). Protein expression was already demonstrated in such vesicles (Teh et al., 2011), paving the way toward artificial cells. However, for membrane diffusion studies, the non-physiological polymer solutions could create artifacts. Another disadvantage is the high amount of lipids that is needed.

## Mechanical micromanipulation of single GUVs

**Figure 13:**
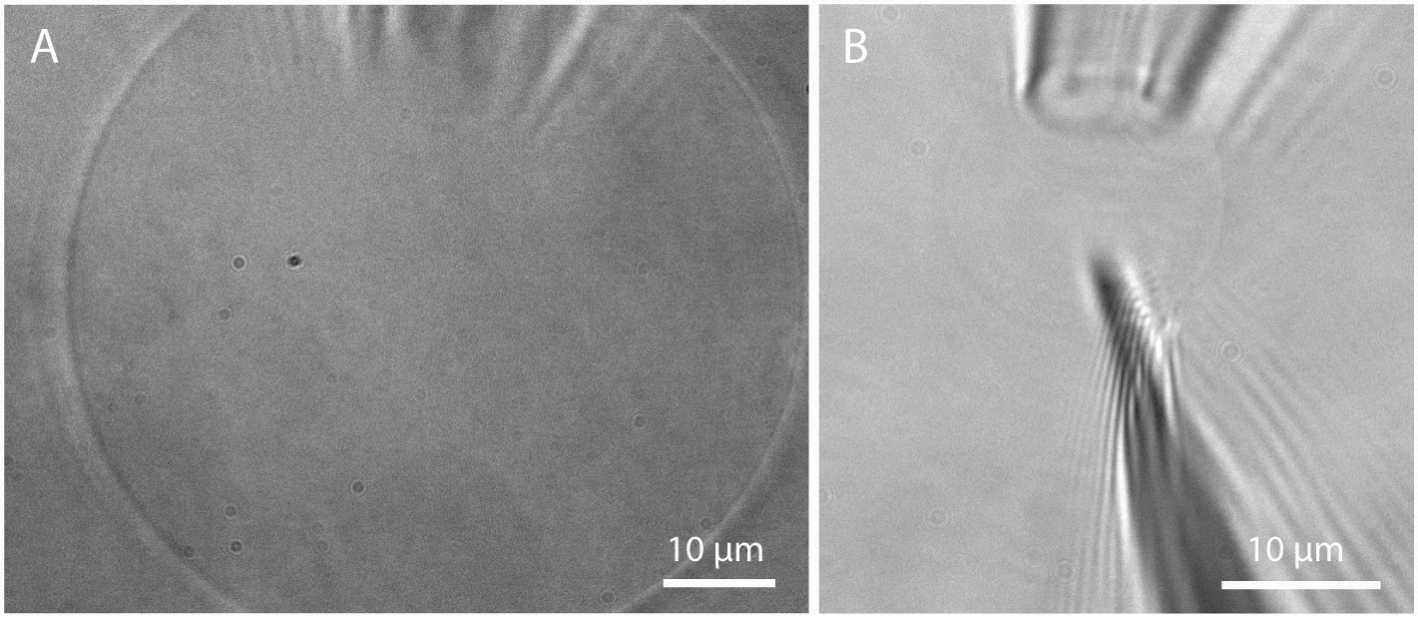
**Micropipette aspiration and injection. (A)** Detached GUV prepared in Bilayer buffer on PVA and aspirated by a micropipette; **(B)** Insertion of a nanopipette (200 nm opening) into an aspirated GUV.

Microscopic studies on single GUVs often require immobilization of free-floating GUVs. The most commonly used method is immobilization on a surface via the biotin-avidin (or streptavidin, neutravidin) binding assay (Kuhn et al., 2012). However, there is evidence that domains in lipid mixtures might be reorganized upon this immobilization process (Sarmento et al., 2012). Trapping in a microfluidic chip by the use of posts (Robinson et al., 2013) offers the possibility to immobilize GUVs without the influence of tethers. For studies requiring a very tight spatial confinement, a more stable trapping can be achieved via micropipette aspiration (Mitchison and Swann, 1954). Here, a glass micropipette with an opening of few μm connected to a micromanipulator is used to aspirate a GUV by the use of a height-adjustable water tank, see Figure 13A. This method is also useful for investigating membrane tension (Tian et al., 2007) and the effect on membrane properties (Portet et al., 2012) but also curvature driven sorting of lipids and proteins (Sorre et al., 2009; Tian and Baumgart, 2009). Going beyond the application of GUVs for studying membrane properties, injection of substances such as proteins, enzymes, or DNA (Angelova et al., 1999; Karlsson et al., 2000) by the use of a nanopipette (demonstrated in Figure 13B) offers another possibility for controlling the biochemical contents of a GUV, taking a step toward artificial cells.

## Conclusion

Electroformation, polymer assisted swelling and microfluidic approaches are the most common methods used to form GUVs for studying basic properties of cellular membranes. Several variations for each method exist together with other approaches such as rapid evaporation (Moscho et al., 1996), local heating (Billerit et al., 2012), reverse emulsion (Pautot et al., 2003a,b) or cDICE (continuous droplet interface crossing encapsulation) (Abkarian et al., 2011; Blosser et al., 2016). Each method has different advantages and disadvantages, which can influence the experiments in various ways.

In order to study the behavior of single lipids in membrane model systems in detail, it is important that the diffusional behavior of the lipids is not altered. To this end, methods such as microfluidic jetting or double emulsion are not ideal because they might leave oil impurities in the membrane. Furthermore, the necessity to use viscous polymer solutions as outer and inner solutions can lead to unwanted artifacts. Nevertheless, these methods allow for the incorporation of proteins or other cellular components into the vesicle or membrane, which can be used for transport through the membrane and as bioreactors.

With electroformation, no solvents are needed and large vesicles can be easily obtained in sucrose solution or water. The formation of GUVs in physiological conditions as well as the incorporation of proteins is still challenging. Furthermore, possible oxidation of lipids during the electroformation process should be kept in mind.

PVA-assisted swelling is possibly the best method to produce GUVs because its impact on the properties of the lipid membrane is negligible, and a high yield of GUVs can be obtained in physiological buffer solutions. GUVs formed by PVA-assisted swelling open many possibilities for studying the lipid membrane. Since it is possible to form large GUVs in physiological buffer solutions over a wide range of lipid composition, the complexity of the lipid membrane can be increased. In this manner, the influence of the surrounding medium and of distinct types of lipids on the overall membrane organization can be studied in a well-controlled model system.

GUVs formed in buffer solutions establish a well-suited model system to study protein-membrane interactions while respecting the necessary salt and pH levels required for protein stability. The formation of GUVs in physiological medium is the initial step to increasing the complexity of model membranes toward mimicking cellular membranes. From the perspective of synthetic biology, *in situ* manipulation and measurements of the contents of individual GUVs can be very valuable for controlled investigations of biochemical reactions in a confined microscopic volume.

## Materials and methods

### Materials

For GUV formation by electroformation and gel-assisted swelling, 1 mg/ml 1,2-dioleoyl-sn-glycero -3-phosphocholine (DOPC) (Avanti Polar Lipids, Alabaster) with 0.02 mol-% 1,2-dioleoyl-sn-glycero-3-phosphoethanolamine (DOPE) labeled with the fluorescent dye Atto-532 (ATTO-TEC, Siegen, Germany) dissolved in chloroform is used. Tris buffer (Tris-EDTA buffer solution, pH 7.4, Sigma-Aldrich, Germany) contains 10 mM Tris-HCl and 1 mM EDTA. Bilayer buffer contains 10 mM Hepes (Gibco Media, USA) and a physiological salt content of 150 mM NaCl (Lonza). PBS (Phosphate buffered saline, Sigma-Aldrich, Germany) contains 150 mM Sodium phosphate and 150 mM NaCl. PVA with a molecular weight of 145,000 from Merck, Germany is used for gel-assisted swelling. For double emulsion, the lipids used are Egg-PC or DOPC. Poly(ethylene glycol) (PEG) and poly(vinyl alcohol) (PVA, molecular weight 40,000) are from Sigma Aldrich, Germany. Squared capillaries with inner diameter of 1.05 mm are purchased from AIT Glass (Rockaway, USA). Round capillaries with outer diameter of 1 mm are from World Precision Instruments Germany, Berlin. PDMS is prepared by mixing Sylgard 184 silicone elastomer base and Sylgard 184 elastomer curing agent (Dow Corning, Wiesbaden, Germany) in 10:1 weight ratio. N-octadecyl-trimethoxy silane (Sigma Aldrich) and 2-(methoxy (polyethyleneoxy) propyl) trimethoxy silane (Gelest Inc., Morrisville, USA) are used for functionalization of the glass capillaries.

### Electroformation

For electroformation on platinum wires, the wires are cleaned by rubbing with kimwipes (Kimberly Clark) with EtOH, rinsing with EtOH and Isopropanol and drying with nitrogen. Three microliter of a 1 mg/ml lipid solution are deposited on each platinum wire with a length of 1 cm and a diameter of 0.35 mm. The plastic lid (vertical geometry) or the ring with the Pt wires (horizontal geometry) is put in a vacuum chamber for at least 2 h. The chamber is assembled by gluing the plastic ring onto a cleaned cover glass (rinsed with EtOH and Isopropanol) by using Picodent Twinsil (Picodent, Wipperfürth, Germany). To avoid bursting of the GUVs on the pure glass, the glass is incubated with 3 mg/ml beta-casein (Sigma Aldrich) solution for 5 min (Garten et al., 2015). After buffer addition, the chambers are connected to a function generator (AFG 21005, ISO-TECH) and an AC voltage is applied for 2 h (for amplitude and frequency see chapter 3). For setting the voltage value, it has to be considered that the resistance between the separated electrodes of the electroformation chamber is very high. Therefore, the output resistance of the function generator should be set to high resistance (1 MΩ).

For GUV formation by an alternating electric field on ITO coated glasses (ITO cover slips with 15–30 Ω, Diamond Coatings, UK), the glasses are subsequently rinsed with EtOH and Isopropanol and dried with a nitrogen stream. 2.5 μl of a 1 mg/ml lipid solution are spread on the conductive site on an area with a diameter of 13 mm. The lipid coated electrodes are put in a vacuum chamber for at least 2 h to evaporate the chloroform completely. A 3 mm thick PDMS spacer, which is smaller than the ITO glasses in one dimension and has an opening of 13 mm, is placed on top of one ITO glass and lined up precisely with one edge of the glass. After filling the opening of the spacer with buffer solution, the second ITO glass with the conductive side facing the lower ITO glass and lined up with the other edge of the spacer is placed on top carefully without leaving air bubbles. Copper tape can be glued onto both ITO glasses at the freestanding area and connected via clamps to a function generator. For good stability, we built a plastic frame with incorporated copper tape connected to wires.

After electroformation, the vesicles are detached from the substrate by lowering the frequency to 2 Hz for 30 min. The free floating vesicles are transferred into an observation chamber by gentle pipetting with a cut tip and rinsing. The observation chamber is coated with 3 mg/ml beta casein solution by incubation for 5 min (Garten et al., 2015).

Electroformation was conducted at room temperature, but the process can be easily adapted to other temperatures (e.g., body temperature) by placing the chamber into a heating block of a dry bath or onto a heat plate in case of flat electrodes. The temperature during the formation process should be well above the transition temperature of all lipids used in the mixture (Morales-Penningston et al., 2010).

### PVA-assisted swelling

To prepare GUVs by gel-assisted swelling, PVA is mixed with water to obtain a 5% (w/w) PVA solution and stirred on a heat plate at around 90°C until the solution is clear. We spin coat 150 μl of 5% PVA for 30 s at a speed of 1200 rpm on a plasma-cleaned cover glass with 25 mm diameter, according to the published protocol (Weinberger et al., 2013). To dry the PVA film, the coated glass is placed for 30 min on a heating plate at 50°C. Five to ten microliter of 1 mg/ml lipid solution are deposited uniformly on the PVA film with a microsyringe. The lipid-coated cover glass is put under vacuum for at least 2 h to evaporate the solvent from the dissolved lipid mixture. To form the chamber, a ring is glued onto the coated cover glass and 1 ml buffer solution is added. After swelling for around 1 h at room temperature, the vesicles are transferred into an observation chamber coated with 3 mg/ml beta casein.

### Double emulsion

Round capillaries are tapered using a fiber puller (Sutter Instruments P-2000, USA). The end of the pulled capillaries is cleaved using a scalpel and polished to create a smooth opening of around 120 and 300 μm, respectively. The 120 μm tapered capillary is made hydrophobic with n-octadecyl-trimethoxy silane by simply holding it for few seconds above the open bottle. The collection capillary is treated with 2-(methoxy (polyethyleneoxy) propyl) trimethoxy silane. For this, acetic acid is mixed to a solution consisting of 95% ethanol and 5% water to adjust the pH to 4.5–5.5. The silane is added (2% final concentration) under stirring for at least 5 min. The capillary is dipped into the solution for 1–2 min and afterwards into ethanol to remove excess material. Subsequently the silane layer is cured for 10 min at 110°C.

After surface functionalization, the capillaries are assembled together via PDMS connectors with a T-junction for flow inlet. The PDMS connectors are created by filling degased PDMS into a form with inserted round and squared capillaries, which are removed after hardening of the PDMS at 65°C for 16 h. T-junctions are created using a puncher. After capillary assembly, tubes are inserted into each PDMS T-junction and connected to an Elveflow pressure control (OB1 MK3, Paris, France). The inner solution consists of 8 wt% PEG and 2 wt% PVA, the outer solution of 10 wt% PVA according to the protocol in Arriaga et al. (2014). The middle phase contains the lipids Egg-PC or DOPC dissolved in 36 vol-% chloroform and 64 vol-% hexane. The double emulsion experiment was performed at room temperature.

### Micropipette aspiration and injection

Glass capillaries with 1 mm outer diameter are pulled into micropipettes with 3–7 μm opening using a fiber puller (Sutter Instruments P-2000, USA). By holding the pipette with a micromanipulator (Sensapex, Finnland) and connecting it to a home-build water tank system, GUVs can be aspirated. Injection is done via a second micropipette with 200 nm tip diameter, which is connected to a second micromanipulator.

### Fluorescence microscopy

For fluorescence imaging, a continuous-wave laser at 532 nm (Verdi 2G, Coherent, USA) is used for illumination. The beam is expanded 4x using a telescope and is focused onto the back focal plane of an oil-immersion objective (60x or 30x, Olympus), creating a wide-field illumination on the sample. The fluorescence light is collected by the objective, reflected by a beam splitter and imaged onto a CCD camera (sensicam qe, PCO, Germany) after passing a 540 nm long pass filter.

## Author contributions

HS and SS performed GUV electroformation and gel-assisted swelling experiments. NB and CW carried out experiments on double emulsion. SS and HS wrote the manuscript. VS supervised the project and revised the manuscript. All authors reviewed and approved the final manuscript for submission.

### Conflict of interest statement

The authors declare that the research was conducted in the absence of any commercial or financial relationships that could be construed as a potential conflict of interest. The handling Editor declared a shared affiliation, though no other collaboration, with several of the authors (HS, SS, VS) and states that the process nevertheless met the standards of a fair and objective review.

## Supplementary material

Movie M1 **DOPC-GUVs after electroformation on ITO-coated glass in 300 mM sucrose and application of an AC field with 2 Hz and 3 Vpp**.

Movie M2 **Swelling of DOPC-GUVs on PVA upon addition of buffer solution and focusing on different planes**. Scale bar: 10 μm.

## References

[B1] Abkarian M. Loiseau E. Massiera G. (2011). Continuous droplet interface crossing encapsulation (cDICE) for high throughput monodisperse vesicle design. Soft Matt. 7, 4610–4614. 10.1039/c1sm05239j

[B2] Aimon S. Manzi J. Schmidt D. Larrosa J. A. Bassereau P. Toombes G. E. S. (2011). Functional reconstitution of a voltage-gated potassium channel in giant unilamellar vesicles. PLoS ONE 6:e25529. 10.1371/journal.pone.0025529 21998666 PMC3188570

[B3] Akashi K. Miyata H. Itoh H. Kinosita K. (1996). Preparation of giant liposomes in physiological conditions and their characterization under an optical microscope. Biophys. J. 71, 3242–3250. 10.1016/S0006-3495(96)79517-6 8968594 PMC1233812

[B4] Angelova M. I. Dimitrov D. S. (1986). Liposome electroformation. Faraday Discuss. Chem. Soc. 81:303 10.1039/dc9868100303

[B5] Angelova M. I. Hristova N. Tsoneva I. (1999). DNA-induced endocytosis upon local microinjection to giant unilamellar cationic vesicles. Eur. Biophys. J. 28, 142–150. 10.1007/s002490050193 10028239

[B6] Angelova M. I. Soléau S. Méléard P. Faucon F. Bothorel P. (1992). Preparation of giant vesicles by external AC electric fields. Kinetics and applications, in Trends in Colloid and Interface Science VI. Progress in Colloid & Polymer Science, Vol. 89, eds Helm C. Lösche M. Möhwald H. (Steinkopff), 127–131.

[B7] Arriaga L. R. Datta S. S. Kim S.-H. Amstad E. Kodger T. E. Monroy F.. (2014). Ultrathin shell double emulsion templated giant unilamellar lipid vesicles with controlled microdomain formation. Small 10, 950–956. 10.1002/smll.201301904 24150883

[B8] Ayuyan A. G. Cohen F. S. (2006). Lipid peroxides promote large rafts: effects of excitation of probes in fluorescence microscopy and electrochemical reactions during vesicle formation. Biophys. J. 91, 2172–2183. 10.1529/biophysj.106.087387 16815906 PMC1557570

[B9] Beauchamp D. L. Khajehpour M. (2012). Studying salt effects on protein stability using ribonuclease t1 as a model system. Biophys. Chem. 161, 29–38. 10.1016/j.bpc.2011.11.004 22197350

[B10] Bi H. Yang B. Wang L. Cao W. Han X. (2013). Electroformation of giant unilamellar vesicles using interdigitated ITO electrodes. J. Mater. Chem. A 1, 7125 10.1039/c3ta10323d

[B11] Billerit C. Jeffries G. D. M. Orwar O. Jesorka A. (2012). Formation of giant unilamellar vesicles from spin-coated lipid films by localized IR heating. Soft Matt. 8, 10823 10.1039/c2sm26394g

[B12] Blosser M. C. Horst B. G. Keller S. L. (2016). cDICE method produces giant lipid vesicles under physiological conditions of charged lipids and ionic solutions. Soft Matt. 12, 7364–7371. 10.1039/C6SM00868B 27510092 PMC5008994

[B13] Böckmann R. A. Hac A. Heimburg T. Grubmüller H. (2003). Effect of sodium chloride on a lipid bilayer. Biophys. J. 85, 1647–1655. 10.1016/S0006-3495(03)74594-9 12944279 PMC1303338

[B14] Bouvrais H. Duelund L. Ipsen J. H. (2014). Buffers affect the bending rigidity of model lipid membranes. Langmuir 30, 13–16. 10.1021/la403565f 24377876

[B15] Coskun Ü. Simons K. (2011). Cell membranes: the lipid perspective. Structure 19, 1543–1548. 10.1016/j.str.2011.10.010 22078554

[B16] Czogalla A. Grzybek M. Jones W. Coskun Ü. (2014). Validity and applicability of membrane model systems for studying interactions of peripheral membrane proteins with lipids. Biochim. Biophys. Acta 1841, 1049–1059. 10.1016/j.bbalip.2013.12.012 24374254

[B17] Dietrich C. Bagatolli L. A. Volovyk Z. N. Thompson N. Levi M. E. Jacobson K.. (2001). Lipid rafts reconstituted in model membranes. Biophys. J. 80, 1417–1428. 10.1016/S0006-3495(01)76114-0 11222302 PMC1301333

[B18] Dimitrov D. S. Angelova M. I. (1987). Lipid swelling and liposome formation on solid surfaces in external electric fields. Prog. Colloid Polym. Sci. 76, 48–56. 10.1007/3-798-50724-4_62

[B19] Doeven M. K. Folgering J. H. A. Krasnikov V. Geertsma E. R. van den Bogaart G. Poolman B. (2005). Distribution, lateral mobility and function of membrane proteins incorporated into giant unilamellar vesicles. Biophys. J. 88, 1134–1142. 10.1529/biophysj.104.053413 15574707 PMC1305118

[B20] Eggeling C. Ringemann C. Medda R. Schwarzmann G. Sandhoff K. Polyakova S.. (2009). Direct observation of the nanoscale dynamics of membrane lipids in a living cell. Nature 457, 1159–1162. 10.1038/nature07596 19098897

[B21] Estes D. J. Mayer M. (2005a). Giant liposomes in physiological buffer using electroformation in a flow chamber. Biochim. Biophys. Acta 1712, 152–160. 10.1016/j.bbamem.2005.03.012 15890312

[B22] Estes D. J. Mayer M. (2005b). Electroformation of giant liposomes from spin-coated films of lipids. Colloids Surf. B Biointer. 42, 115–123. 10.1016/j.colsurfb.2005.01.016 15833662

[B23] Fenz S. F. Merkel R. Sengupta K. (2009). Diffusion and intermembrane distance: case study of avidin and e-cadherin mediated adhesion. Langmuir 25, 1074–1085. 10.1021/la803227s 19072315

[B24] Fenz S. F. Sengupta K. (2012). Giant vesicles as cell models. Integr. Biol. 4, 982–995. 10.1039/c2ib00188h 22829218

[B25] Funakoshi K. Suzuki H. Takeuchi S. (2007). Formation of giant lipid vesiclelike compartments from a planar lipid membrane by a pulsed jet flow. J. Am. Chem. Soc. 129, 12608–12609. 10.1021/ja074029f 17915869

[B26] Garten M. Aimon S. Bassereau P. Toombes G. E. (2015). Reconstitution of a transmembrane protein, the voltage-gated ion channel, KvAP, into giant unilamellar vesicles for microscopy and patch clamp studies. J. Visual. Exp. 95:52281 10.3791/52281 PMC4354550 25650630

[B27] Good N. E. Winget G. D. Winter W. Connolly T. N. Izawa S. Singh R. M. (1966). Hydrogen ion buffers for biological research. Biochemistry 5, 467–477. 10.1021/bi00866a011 5942950

[B28] Heinemann F. Schwille P. (2011). Preparation of micrometer-sized free-standing membranes. Chemphyschem 12, 2568–2571. 10.1002/cphc.201100438 21809429

[B29] Hennesthal C. Drexler J. Steinem C. (2002). Membrane-suspended nanocompartments based on ordered pores in alumina. Chemphyschem 3, 885–889. 10.1002/1439-7641(20021018)3:10<885::AID-CPHC885>3.0.CO;2-9 12465188

[B30] Hennesthal C. Steinem C. (2000). Pore-spanning lipid bilayers visualized by scanning force microscopy. J. Am. Chem. Soc. 122, 8085–8086. 10.1021/ja000940j

[B31] Herold C. Chwastek G. Schwille P. Petrov E. P. (2012). Efficient electroformation of supergiant unilamellar vesicles containing cationic lipids on ITO-coated electrodes. Langmuir 28, 5518–5521. 10.1021/la3005807 22424289

[B32] Honigmann A. Walter C. Erdmann F. Eggeling C. Wagner R. (2010). Characterization of horizontal lipid bilayers as a model system to study lipid phase separation. Biophys. J. 98, 2886–2894. 10.1016/j.bpj.2010.03.033 20550901 PMC2884238

[B33] Horger K. S. Estes D. J. Capone R. Mayer M. (2009). Films of agarose enable rapid formation of giant liposomes in solutions of physiologic ionic strength. J. Am. Chem. Soc. 131, 1810–1819. 10.1021/ja805625u 19154115 PMC2757642

[B34] Hsieh C.-L. Spindler S. Ehrig J. Sandoghdar V. (2014). Tracking single particles on supported lipid membranes: multimobility diffusion and nanoscopic confinement. J. Phys. Chem. B 118, 1545–1554. 10.1021/jp412203t 24433014

[B35] Jørgensen I. L. Kemmer G. C. Pomorski T. G. (2016). Membrane protein reconstitution into giant unilamellar vesicles: a review on current techniques. Eur. Biophys. J. [Epub ahead of print]. 10.1007/s00249-016-1155-9 27437691

[B36] Karlsson M. Nolkrantz K. Davidson M. J. Strömberg A. Ryttsén F. Akerman B.. (2000). Electroninjection of colloid particles and biopolymers into single unilamellar liposomes and cells for bioanalytical applications. Anal. Chem. 72, 5857–5862. 10.1021/ac0003246 11128948

[B37] Kirchner S. R. Ohlinger A. Pfeiffer T. Urban A. S. Stefani F. D. Deak A.. (2012). Membrane composition of jetted lipid vesicles: a Raman spectroscopy study. J. Biophotonics 5, 40–46. 10.1002/jbio.201100058 22147675

[B38] Koerner M. M. Palacio L. A. Wright J. W. Schweitzer K. S. Ray B. D. Petrache H. I. (2011). Electrodynamics of lipid membrane interactions in the presence of zwitterionic buffers. Biophys. J. 101, 362–369. 10.1016/j.bpj.2011.05.062 21767488 PMC3136761

[B39] Kuhn P. Eyer K. Robinson T. Schmidt F. I. Mercer J. Dittrich P. S. (2012). A facile protocol for the immobilisation of vesicles, virus particles, bacteria and yeast cells. Integr. Biol. 4, 1550–1555. 10.1039/c2ib20181j 23147942

[B40] Kusumi A. Sako Y. Yamamoto M. (1993). Confined lateral diffusion of membrane receptors as studied by single particle tracking (nanovid Microscopy). Effects of calcium-induced differentiation in cultured epithelial cells. Biophys. J. 65, 2021–2040. 10.1016/S0006-3495(93)81253-0 8298032 PMC1225938

[B41] Ladokhin A. S. Fernández-Vidal M. White S. H. (2010). CD spectroscopy of peptides and proteins bound to large unilamellar vesicles. J. Membr. Biol. 236, 247–253. 10.1007/s00232-010-9291-0 20706833 PMC2938439

[B42] Larsen J. Hatzakis N. S. Stamou D. (2011). Observation of inhomogeneity in the lipid composition of individual nanoscale liposomes. J. Am. Chem. Soc. 133, 10685–10687. 10.1021/ja203984j 21688773

[B43] Li W. Wang Q. Yang Z. Wang W. Cao Y. Hu N. (2016). Impacts of electrical parameters on the electroformation of giant vesicles on ITO glass chips. Colloids Surf. B Biointer. 1, 560–566. 10.1016/j.colsurfb.2015.11.020 26628330

[B44] López Mora N. L. Hansen J. S. Gao Y. Ronald A. A. Kieltyka R. Malmstadt N.. (2014). Preparation of size tunable giant vesicles from cross-linked dextran(ethylene glycol) hydrogels. Chem. Commun. 50, 1953–1955. 10.1039/c3cc49144g 24407820 PMC4114216

[B45] Lide D. R. (2005). CRC Handbook of Chemistry and Physics, 92nd Edn. Boca Raton, FL: Haynes, W. M.

[B46] Lira R. B. Dimova R. Riske K. A. (2014). Giant unilamellar vesicles formed by hybrid films of agarose and lipids display altered mechanical properties. Biophys. J. 107, 1609–1619. 10.1016/j.bpj.2014.08.009 25296313 PMC4190656

[B47] Micheletto Y. M. Marques C. M. Silveira N. P. Schroder A. P. (2016). Electroformation of giant unilamellar vesicles: investigating vesicle fusion versus bulge merging. Langmuir 32, 8123–8130. 10.1021/acs.langmuir.6b01679 27409245

[B48] Mitchison J. M. Swann M. M. (1954). The mechanical properties of the cell surface. J. Exp. Biol. 31, 443–460.

[B49] Montes L. R. Alonso A. Goni F. M. Bagatolli L. A. (2007). Giant unilamellar vesicles electroformed from native membranes and organic lipid mixtures under physiological conditions. Biophys. J. 93, 3548–3554. 10.1529/biophysj.107.116228 17704162 PMC2072068

[B50] Morales-Penningston N. F. Wu J. Farkas E. R. Goh S. L. Konyakhina T. M. Zheng J. Y.. (2010). GUV preparation and imaging: minimizing artefacts. Biochim. Biophys. Acta 1798, 1324–1332. 10.1016/j.bbamem.2010.03.011 20302841 PMC2885611

[B51] Moscho A. Orwar O. Chiu D. T. Modi B. P. Zare R. N. (1996). Rapid preparation of giant unilamellar vesicles. Proc. Natl. Acad. Sci. U.S.A. 93, 11443–11447. 10.1073/pnas.93.21.11443 8876154 PMC38076

[B52] Mueller P. Rudin D. O. Tien H. T. Wescott W. C. (1962). Reconstitution of cell membrane structure *in vitro* and its transformation into an excitable system. Nature 194, 979–980. 10.1038/194979a0 14476933

[B53] Mueller V. Ringemann C. Honigmann A. Schwarzmann G. Medda R. Leutenegger M.. (2011). STED Nanoscopy reveals molecular details of cholesterol- and cytoskeleton modulated lipid interactions in living cells. Biophys. J. 101, 1651–1660. 10.1016/j.bpj.2011.09.006 21961591 PMC3183802

[B54] Niri V. H. Flatt B. K. Fakhraai Z. Forrest J. A. (2009). Simultaneous monitoring of electroformation of phospholipid vesicles by quartz crystal microbalance and optical microscopy. Chem. Phys. Lipids 163, 36–41. 10.1016/j.chemphyslip.2009.10.004 19883636

[B55] Pautot S. Frisken B. J. Weitz D. A. (2003a). Production of unilamellar vesicles using an inverted emulsion. Langmuir 19, 2870–2879. 10.1021/la026100v

[B56] Pautot S. Frisken B. J. Weitz D. A. (2003b). Engineering asymmetric vesicles. Proc. Natl. Acad. Sci. U.S.A. 100, 10718–10721. 10.1073/pnas.1931005100 12963816 PMC196870

[B57] Phillips R. Kondev J. Theriot J. Garcia H. G. Orme N. (2013). Physical Biology of the Cell, 2nd Edn. London; New York, NY: Garland Science.

[B58] Politano T. J. Froude V. E. Jing B. Zhu Y. (2010). AC-electric field dependent electroformation of giant lipid vesicles. Colloids Surf. B, Biointer. 79, 75–82. 10.1016/j.colsurfb.2010.03.032 20413284

[B59] Portet T. Gordon S. E. Keller S. L. (2012). Increasing membrane tension decreases miscibility temperatures; an experimental demonstration via micropipette aspiration. Biophys. J. 103, L35–37. 10.1016/j.bpj.2012.08.061 23083725 PMC3475388

[B60] Pott T. Bouvrais H. Méléeard P. (2008). Giant unilamellar vesicle formation under physiologically relevant conditions. Chem. Phys. Lipids 154, 115–119. 10.1016/j.chemphyslip.2008.03.008 18405664

[B61] Przybylo M. Sýkora J. Humpolíckova J. Benda A. Zan A. Hof M. (2006). Lipid diffusion in giant unilamellar vesicles is more than 2 times faster than in supported phospholipid bilayers under identical conditions. Langmuir 22, 9096–9099. 10.1021/la061934p 17042516

[B62] Quemeneur F. Sigurdsson J. K. Renner M. Atzberger P. J. Bassereau P. Lacoste D. (2014). Shape matters in protein mobility within membranes. Proc. Natl. Acad. Sci. U.S.A. 111, 5083–5087. 10.1073/pnas.1321054111 24706877 PMC3986167

[B63] Reeves J. P. Dowben R. M. (1969). Formation and properties of thin-walled phospholipid vesicles. J. Cell. Physiol. 73, 49–60. 10.1002/jcp.1040730108 5765779

[B64] Richmond D. L. Schmid E. M. Martens S. Stachowiak J. C. Liska N. Fletcher D. A. (2011). Forming giant vesicles with controlled membrane composition, asymmetry, and contents. Proc. Natl. Acad. Sci. U.S.A. 108, 9431–9436. 10.1073/pnas.1016410108 21593410 PMC3111313

[B65] Robinson T. Kuhn P. Eyer K. Dittrich P. S. (2013). Microfluidic trapping of giant unilamellar vesicles to study transport through a membrane pore. Biomicrofluidics 7:044105. 10.1063/1.4816712 24404039 PMC3739824

[B66] Rodriguez N. Pincet F. Cribier S. (2005). Giant vesicles formed by gentle hydration and electroformation: a comparison by fluorescence microscopy. Colloids Surf. B Biointer. 42, 125–130. 10.1016/j.colsurfb.2005.01.010 15833663

[B67] Roux A. Koster G. Lenz M. Sorre B. Manneville J.-B. Nassoy P.. (2010). Membrane curvature controls dynamin polymerization. Proc. Natl. Acad. Sci. U.S.A. 107, 4141–4146. 10.1073/pnas.0913734107 20160074 PMC2840091

[B68] Runas K. A. Malmstadt N. (2015). Low levels of lipid oxidation radically increase the passive permeability of lipid bilayers. Soft Matt. 11, 499–505. 10.1039/C4SM01478B 25415555 PMC4477792

[B69] Sankhagowit S. Wu S.-H. Biswas R. Riche C. T. Povinelli M. L. Malmstadt N. (2014). The dynamics of giant unilamellar vesicle oxidation probed by morphological transitions. Biochim. Biophys. Acta 1838, 2615–2624. 10.1016/j.bbamem.2014.06.020 24998358

[B70] Sarmento M. J. Prieto M. Fernandes F. (2012). Reorganization of lipid domain distribution in giant unilamellar vesicles upon immobilization with different membrane tethers. Biochim. Biophys. Acta 1818, 2605–2615. 10.1016/j.bbamem.2012.05.028 22664063

[B71] Sezgin E. Gutmann T. Buhl T. Dirkx R. Grzybek M. Coskun Ü.. (2015). Adaptive lipid packing and bioactivity in membrane domains. PLoS ONE 10:e0123930. 10.1371/journal.pone.0123930 25905447 PMC4408024

[B72] Shum H. C. Lee D. Yoon I. Kodger T. Weitz D. A. (2008). Double emulsion templated monodisperse phospholipid vesicles. Langmuir 24, 7651–7653. 10.1021/la801833a 18613709

[B73] Sorre B. Callan-Jones A. Manneville J. B. Nassoy P. Joanny J. F. Prost J.. (2009). Curvature-driven lipid sorting needs proximity to a demixing point and is aided by proteins. Proc. Natl. Acad. Sci. U.S.A. 106, 5622–5626. 10.1073/pnas.0811243106 19304798 PMC2667082

[B74] Stachowiak J. C. Richmond D. L. Li T. H. Brochard-Wyart F. Fletcher D. A. (2009). Inkjet formation of unilamellar lipid vesicles for cell-like encapsulation. Lab Chip 9, 2003–2009. 10.1039/b904984c 19568667 PMC2937252

[B75] Tareste D. Shen J. Melia T. J. Rothman J. E. (2008). SNAREpin/Munc18 promotes adhesion and fusion of large vesicles to giant membranes. Proc. Natl. Acad. Sci. U.S.A. 105, 2380–2385. 10.1073/pnas.0712125105 18268324 PMC2268145

[B76] Teh S.-Y. Khnout R. Fan H. Lee A. P. (2011). Stable, biocompaticle lipid vesicle generation by solvent extraction-based droplet microfluidics. Biomicrofluidics 5:044113. 10.1063/1.3665221 22685501 PMC3368830

[B77] Tian A. Baumgart T. (2009). Sorting of lipids and proteins in membrane curvature gradients. Biophys. J. 96, 2676–2688. 10.1016/j.bpj.2008.11.067 19348750 PMC2711293

[B78] Tian A. Johnson C. Wang W. Baumgart T. (2007). Line tension at fluid membrane domain boundaries measured by micropipette aspiration. Phys. Rev. Lett. 98:208102. 10.1103/physrevlett.98.208102 17677743

[B79] Tsai F.-C. Stuhrmann B. Koenderink G. H. (2011). Encapsulation of active cytoskeletal protein networks in cell-sized liposomes. Langmuir 27, 10061–10071. 10.1021/la201604z 21707043

[B80] Tsumoto K. Matsuo H. Tomita M. Yoshimura T. (2009). Efficient formation of giant liposomes through the gentle hydration of phosphatidylcholine films doped with sugar. Colloids Surf. B Biointer. 68, 98–105. 10.1016/j.colsurfb.2008.09.023 18993037

[B81] van den Bogaart G. Hermans N. Krasnikov V. de Vries A. H. Poolman B. (2007). On the decrease in lateral mobility of phospholipids by sugars. Biophys. J. 92, 1598–1605. 10.1529/biophysj.106.096461 17142271 PMC1796821

[B82] van Swaay D. deMello A. (2013). Microfluidic methods for forming liposomes. Lab Chip 13, 752–767. 10.1039/c2lc41121k 23291662

[B83] Veatch S. L. Polozov I. V. Gawrisch K. Keller S. L. (2004). Liquid domains in vesicles investigated by NMR and fluorescence microscopy. Biophys. J. 86, 2910–2922. 10.1016/S0006-3495(04)74342-8 15111407 PMC1304159

[B84] Wagner M. L. Tamm L. K. (2000). Tethered polymer-supported planar lipid bilayers for reconstitution of integral membrane proteins: silane-polyethyleneglycol-lipid as a cushion and covalent linker. Biophys. J. 79, 1400–1414. 10.1016/S0006-3495(00)76392-2 10969002 PMC1301034

[B85] Walde P. Cosentino K. Engel H. Stano P. (2010). Giant vesicles: preparations and applications. Chembiochem 11, 848–865. 10.1002/cbic.201000010 20336703

[B86] Wang Y.-H. Collins A. Guo L. Smith-Dupont K. B. Gai F. Svitkina T.. (2012). Divalent cation-induced cluster formation by polyphosphoinositides in model membranes. J. Am. Chem. Soc. 134, 3387–3395. 10.1021/ja208640t 22280226 PMC3445022

[B87] Weinberger A. Tsai F.-C. Koenderink G. H. Schmidt T. F. Itri R. Meier W.. (2013). Gel-assisted formation of giant unilamellar vesicles. Biophys. J. 105, 154–164. 10.1016/j.bpj.2013.05.024 23823234 PMC3699747

[B88] Wieprecht T. Apostolov O. Beyermann M. Seelig J. (2000). Membrane binding and pore formation of the antibacterial peptide PGLa: thermodynamic and mechanistic aspects. Biochemistry 39, 442–452. 10.1021/bi992146k 10631006

[B89] Zhou Y. Berry C. K. Storer P. A. Raphael R. M. (2007). Peroxidation of polyunsaturated phosphatidyl-choline lipids during electroformation. Biomaterials 28, 1298–1306. 10.1016/j.biomaterials.2006.10.016 17107709

